# Antioxidative 1,4-Dihydropyridine Derivatives Modulate Oxidative Stress and Growth of Human Osteoblast-Like Cells In Vitro

**DOI:** 10.3390/antiox7090123

**Published:** 2018-09-19

**Authors:** Lidija Milkovic, Tea Vukovic, Neven Zarkovic, Franz Tatzber, Egils Bisenieks, Zenta Kalme, Imanta Bruvere, Zaiga Ogle, Janis Poikans, Astrida Velena, Gunars Duburs

**Affiliations:** 1 Laboratory for Oxidative Stress, Rudjer Boskovic Institute, Bijenicka 54, 10000 Zagreb, Croatia; Lidija.Milkovic@irb.hr (L.M.); Tea.Vukovic@irb.hr (T.V.); 2 Institute of Pathophysiology and Immunology, Medical University of Graz, A-8036 Graz, Austria; franz@tatzber.at; 3 Latvian Institute of Organic Synthesis, 21 Aizkraukles Str., LV-1006 Riga, Latvia; Egils.Bisenieks@osi.lv (E.B.); kalme@osi.lv (Z.K.); Imanta.Bruvere@gmail.com (I.B.); zaiga.ogle@osi.lv (Z.O.); japo@osi.lv (J.P.); gduburs@osi.lv (G.D.)

**Keywords:** 1,4-dihydropyridine(s) (DHPs), oxidative stress, reactive oxygen species (ROS), antioxidant (AO), antioxidative activity (AOA), glutathione, cell viability and proliferation, 4-hydroxynonenal (4-HNE)

## Abstract

Oxidative stress has been implicated in pathophysiology of different human stress- and age-associated disorders, including osteoporosis for which antioxidants could be considered as therapeutic remedies as was suggested recently. The 1,4-dihydropyridine (DHP) derivatives are known for their pleiotropic activity, with some also acting as antioxidants. To find compounds with potential antioxidative activity, a group of 27 structurally diverse DHPs, as well as one pyridine compound, were studied. A group of 11 DHPs with 10-fold higher antioxidative potential than of uric acid, were further tested in cell model of human osteoblast-like cells. Short-term combined effects of DHPs and 50 µM H_2_O_2_ (1-h each), revealed better antioxidative potential of DHPs if administered before a stressor. Indirect 24-h effect of DHPs was evaluated in cells further exposed to mild oxidative stress conditions induced either by H_2_O_2_ or tert-butyl hydroperoxide (both 50 µM). Cell growth (viability and proliferation), generation of ROS and intracellular glutathione concentration were evaluated. The promotion of cell growth was highly dependent on the concentrations of DHPs used, type of stressor applied and treatment set-up. Thiocarbatone **III-1**, E2-134-1 **III-4**, Carbatone **II-1**, AV-153 **IV-1**, and Diethone **I** could be considered as therapeutic agents for osteoporosis although further research is needed to elucidate their bioactivity mechanisms, in particular in respect to signaling pathways involving 4-hydroxynoneal and related second messengers of free radicals.

## 1. Introduction

Oxidative stress, defined as an imbalance between pro-oxidants and antioxidants in the favor of the former [1], has been implicated in the pathogenesis of numerous stress- and age-associated diseases, whether as a cause or as a consequence of respective illness progression [2]. The consensus among researchers in this field highlights the need of its better understanding as well as balancing oxidative homeostasis to the levels that promote health [3]. Indeed, antioxidants per se or drugs with antioxidative properties are important for reducing the detrimental levels of reactive oxygen species (ROS). Yet, the importance of ROS-related redox signaling in normal cellular maintenance should not be neglected, nor the fact that antioxidative and/or pro-oxidative activity are in the background of (un)desirable activities of many drugs and physiologically active compounds [3,4,5]. Thus, studies revealing bioactivities of natural antioxidants are complemented by scientific efforts aiming to synthetize new bioactive substances with antioxidant features that could help maintaining oxidative homeostasis of the living cells.

1,4-Dihydropyridine derivatives (DHPs) are a group of pleiotropic physiologically active compounds (reviewed in Swarnalatha et al. [6] and Velena et al. [7]), among which many 4-nitrophenyl- and differently substituted DHPs are known as effective antihypertensive agents. While condensed DHPs were suggested as agents that affect stem cell differentiation [8], several DHPs exert anti- or pro-oxidant effects in various systems both in vitro as well as in vivo [7,9].

Our present article extends the knowledge about antioxidative and potentially pro-oxidative activities of the well-known, water-insoluble antioxidant Diethone **I** (also known as Dietone, Diludine, Hantzsch ester, HEH) [7], in comparison to the water-soluble antimutagenic [10] and antimetastatic [11] compound Carbatone (disodium-2,6-dimethyl-1,4-dihydropyridine-3,5-bis(carbonyloxyacetate)) **II-1**, which prevents DNA lesions [12,13,14,15]. Moreover, comparison was done also with their analogues and with novel DHPs (see Table 1, compounds mentioned as MM) and their derivatives.

Therefore, a set of 27 structurally diverse (of monocyclic as well as of condensed ring structures, having symmetric as well as asymmetric alkyl-, alkoxyalkyl-, aryl-, aralkyl- or heteryl- substituents on positions 2, 3, 4, 5 and 6) synthetic 1,4-dihydropyridine compounds (as well as for comparison one pyridine type compound, an analogue (oxidized form) of diamide analogue J-12-25 **V**) (see Table 1 below) of which 14 are water soluble DHP compounds (including antimutagenic, DNA protecting, antimetastatic, antiischaemic) and 14 are water-insoluble (more lipid soluble): 13 DHP compounds (including antioxidant and radioprotector Hantzsch ester Diethone (Dietone) **I**) and one pyridine analogue were studied.

While possibly beneficial effects of antioxidants are mostly considered for patients suffering from malignant, cardiovascular, neurodegenerative and inflammatory diseases, osteoporosis is an age-related disease characterized by bone loss due to the impaired bone formation and increased bone resorption, which is less often considered as an oxidative stress-associated disease. However, requests for treatment strategies of this chronic disease that would promote bone formation [16] also point to oxidative stress as an important factor in pathogenesis of osteoporosis [17,18], consequently highlighting antioxidants as important factors for prevention and treatment of osteoporosis [17,19].

Therefore, in our study, we used human osteoblast-like cells (HOS cell line) which are well known as a model of human osteoblasts [20,21,22] aiming to reveal DHPs that can act as antioxidant(s) in cell-free systems (Total antioxidative capacity (TAC) and Total oxidative capacity (TOC) assays, respectively) but could also affect viability and growth of HOS cells under mild oxidative stress induced by two different stressors (hydrogen peroxide and tert-butyl hydroperoxide).

## 2. Materials and Methods

### 2.1. Compounds

The studied 27 DHP compounds belong to several (five, **I**–**V**, see Table 1) relative types. All tested DHPs were synthesized at the Latvian Institute of Organic Synthesis [24,25,26,27,28,29,30]. Already described compounds are referenced in Table 1; new compounds have been synthesized by making use of methods indicated below for each type and indexed as MM (*MM) in Table 1.

Dietone **I** is the basic most studied compound, all studied types are its derivatives. It could be used as standard. It has been elaborated and developed, used as radioprotector, antioxidant to preserve carotene, vitamins A and E, growth stimulant. Dietone **I** was synthesized according to modified Hantzsch syntesis: heterocyclization of acetoacetic ester and urotropine [23]. **Type II** compounds (**II-1**–**II-6**) or Carbatone **II-1** and its derivatives were prepared by making use of 1 or 2 equivalents of ethoxycarbonylmethyl ester of acetoacetic acid instead of or as addition to acetoacetic ester. For synthesis of soluble in water salts mild hydrolysis of distant ester group was performed. Compounds (**II-1**–**II-6**) were obtained according to procedure described in patents [24,25,26]. **Type III** compounds (**III-1**–**III-12**) or Thiocarbatone **III-1** and its derivatives were prepared by making use of one or two equivalents of ethoxycarbonylmethyl ester of acetothioacetic acid instead of or as addition to acetoacetic ester. Again, for synthesis of soluble in water salts mild hydrolysis of distant ester group was performed. Compounds (**III-1**–**III-12**) were obtained following the reported method [26].

**Type IV** compounds (derivatives of 1,4-dihydroisonicotinic acid **IV-1**–**IV-8**) were synthesized by making use of glyoxylic acid or its ester as aldehyde part, ethyl or methyl ester of acetoacetic acid (and/or additionally ethoxycarbonylmethyl ester of acetylacetic acid) and ammonia. AV-153 (**IV-1**) can be as intermediate for synthesis of Glutapyrone (**IV-8**) [28]. Compounds (MM, **IV**-**1**–**IV-6**) were obtained according to procedure described in [27]. Pyridine derivative J-12-25 (**V**) was obtained via oxidation of related DHP with sodium nitrite in acetic acid.

Major features of the substances tested are presented in Table 1.

Fresh stock solutions of DHPs were prepared in adequate solvent (listed in Table 1) in a concentration of 10 mg/mL prior to each experiment.

### 2.2. Total Antioxidative Capacity (TAC) Assay

The total antioxidative capacity assay relying on the ability of tested compounds to scavenge hydrogen peroxide, thus, competing with peroxidase and preventing oxidation of a chromogenic substrate tetramethylbenzidine (TMB), was performed as previously described [31], with a slight modification as described. The scavenging ability of DHP compounds was compared to ranging concentration of uric acid serving as standard antioxidant. Briefly, 25 μL of a standard (uric acid serial dilutions from 0–12 mM) or a sample (27 DHPs, as well one pyridine type compound) each 10 mg/mL) were pipetted into each well of the 96-well microplate and mixed with 100 µL Reagent A (0.1 M citric buffer containing 0.03% (*v*/*v*) hydrogen-peroxide). The first absorbance was measured at 450 nm (Multiscan Ex, Thermo Electron Corporation, Shanghai, China) followed by the addition of 50 µL Reagent B containing 1.25 mU horse radish peroxidase (HRP; Sigma Aldrich, St. Louis, MO, USA) and 0.416 mM TMB (Sigma Aldrich, St. Louis, MO, USA) in citric buffer. After 15-min incubation time, reaction was stopped with 2 M H_2_SO_4_ and the second absorbance was measured at 450 nm (Multiscan Ex, Thermo Electron Corporation, Shanghai, China). The difference between the second and the first absorbance values of the tested compounds was interpolated from the uric acid standard curve and results are presented as mM Uric acid equivalent.

### 2.3. Total Oxidative Capacity (TOC) Assay

A total oxidative capacity assay was performed as previously described [32]. The assay determines peroxides present in the sample by their reaction with peroxidase followed by a color reaction with chromogenic substrate TMB. Quantification is achieved by serial dilutions of a standard hydrogen peroxide solution. Briefly, 10 μL of a standard (hydrogen peroxide serial dilutions from 0–0.9791 mM) or a sample (27 DHPs, as well one pyridine type compound) was pipetted into each well of the 96-well microplate, mixed with 200 µL of reaction mixture (25 mU HRP, TMB, and substrate buffer in a proportion of 1:10:100 (*v*:*v*:*v*)) and initial absorbance at 450 nm was measured (Multiscan Ex, Thermo Electron Corporation, Shanghai, China). Plate was further incubated for 20 min and the second absorbance at 450 nm was measured after stopping the reaction with 2 M H_2_SO_4_. The results are expressed as µM H_2_O_2_ hydrogen peroxide equivalent.

### 2.4. Cell Culture and Treatments

The human osteosarcoma cell line HOS (ATCC^®^ CRL-1543™), purchased from the American Type Culture Collection (ATCC, LGC Standards GmbH, Wesel, Germany) was used as an osteoblast model to test possible the in vitro effects of selected compounds. The cells were cultivated in T75 cell culture flasks (TPP, Trasadingen, Switzerland) in Dulbecco’s Modified Eagle’s Medium (DMEM) with 10% (*v*/*v*) fetal calf serum (FCS) at 37 °C in humidified atmosphere with 5% CO_2_. Prior to experiments, cells were harvested with 0.25% (*w*/*v*) Trypsin-0.53 mM EDTA (Ethylenediaminetetraacetic acid) solution and counted with Trypan Blue Exclusion Assay in Bürker-Türk hemocytometer (Brand, Wertheim, Germany). Thus prepared cells were seeded at a density of 2 × 10^4^ cells/well into 96-microwell plates (TPP, Trasadingen, Switzerland) and left for 24 h to attach. Afterwards, we evaluated the potential of selected compounds to influence intracellular ROS production upon administration of hydrogen peroxide. Cell cultures were either first exposed to 50 µM hydrogen peroxide for one hour before addition of selected compounds (100 or 1000 µM) or vice versa. The immediate effect on cellular viability was also evaluated in case of cells that were first exposed to selected compounds for one hour, followed by one-hour incubation with hydrogen peroxide, aiming to test possible beneficial effects of the tested substances that might prevent the onset of the cellular oxidative stress.

The second series of experiments were conducted with prolonged (24-h) exposure of cells to selected compounds before one-hour exposure to hydrogen-peroxide. The experiments are explained in more details in the following subsections.

### 2.5. Cellular Viability (Tetrazolium Reduction Assay)

Viability (metabolic activity) of the cultured HOS cells was evaluated with the EZ4U assay (Biomedica, Wien, Austria) according to manufacturer’s instructions. The assay principle is based on reduction of tetrazolium salts to colored formazan derivatives by living cells, the intensity of which is measured spectrophotometrically as proportional to the number of viable cells in the culture sample.

Cells seeded at a density of 2 × 10^4^ cells/well in 96-microwell plates (TPP, Trasadingen, Switzerland) were left to attach for 24 h prior to treatments. For the short-term treatment, cells were first treated with selected compounds (100 µM and 1000 µM concentration; except for Diethone **I** for which only 100 µM concentration was used because it precipitates at 1000 µM) for 1 h, after which hydrogen peroxide was added for an additional hour. In case of experiments with longer exposure to selected compounds, HOS cells were treated with different concentrations of selected compounds (ranging from 10 µM to 1000 µM, depending on the compound used) and left for additional 24 h, followed by the change of cell culture medium. Cellular viability was determined for cell culture either treated just by selected compounds or for those additionally treated with 50 µM hydrogen peroxide or with 50 µM tert-butyl hydroperoxide (tBHP; Sigma Aldrich, St. Louis, MO, USA) for one-hour, respectively. Afterwards, the medium was replaced with 200 µL Hanks’ solution (pH 7.4) and 20 µL of dye solution was added to each well followed by two-hour incubation at 37 °C. The absorbance was measured at 450 nm with a reference wavelength of 620 nm using a microplate reader (Multiscan Ex, Thermo Electron Corporation, Shanghai, China). Thus obtained results were expressed as percentage of non-treated control.

### 2.6. Cell Proliferation (BrdU Assay)

Cell proliferation was determined in the experiments where cells were exposed to selected DHP compounds for 24-h used alone or in combination with 50 µM hydrogen peroxide or tBHP, as described in the previous section. The 5-bromo-2′-deoxyuridine (BrdU) colorimetric assay (Roche Applied Science, Mannheim, Germany) was used, according to the manufacturer’s instruction. The assay relies on the ability of the BrdU to incorporate into cellular DNA during proliferation which is further detected by an anti-BrdU antibody. Briefly, after specified time points of treatments (24-h treatment with the compound alone or followed by 1-h treatment with hydrogen peroxide and tBHP), the BrdU was added to each well and left for two-hours at 37 °C. The culture medium was removed and the cells were washed twice with Hanks’ solution before fixation with FixDenat solution for 30 min. Subsequently, the anti-BrdU-peroxidase (1:100) was added to each well and incubated at room temperature for 90 min. After removing the unbound antibody conjugate, 100 μL of the substrate solution was added and allowed to stand for 15 min. The reaction was quenched by adding 25 μL of a 1 M H_2_SO_4_ solution. The absorbance was measured at 450 nm with a reference wavelength of 620 nm using a microplate reader (Multiscan Ex, Thermo Electron Corporation, Shanghai, China).The results are presented as percentage of non-treated control.

### 2.7. Measurement of intracellular ROS production

The ROS measurement is based on the intracellular oxidation of 2′,7′-dichlorodihydrofluorescein diacetate (DCFH-DA; Sigma-Aldrich, St. Louis, MO, USA) to a fluorescent 2′,7′-dichlorofluorescein (DCF) that can be measured. Intracellular ROS levels were measured upon short-term (1-h) treatment and after 24-h treatment with selected DHPs, described hereafter. For all experiments, HOS cells were seeded at the density of 2 × 10^4^ cells/well into white 96-microwell plates (Thermo Fisher Scientific, Nunc A/S, Roskilde, Denmark) and left for 24 h to attach.

In the short-term treatment, time-dependent ROS production was measured in cells treated at specific time points as follows: (a) hydrogen peroxide 1-h + DHPs, or (b) DHPs 1-h + hydrogen peroxide. Cells were first incubated with 10 μM DCFH-DA at 37 °C for 30 min, followed by fresh media exchange and a zero-point ROS measurement with a Cary Eclipse Fluorescence Spectrophotometer (Varian Australia Pty Ltd, Mulgrave, Victoria, Australia) with excitation at 500 nm and emission detection at 530 nm. Treatments were carried out with 1000 µM concentration of DHPs (exception Diethone **I** used as 100 µM because it precipitates at 1000 µM) and 50 µM hydrogen peroxide without the medium change. Further fluorescence measurements were done: immediately after addition of the first treatment (5-min point), 30- and 60-min points, immediately after addition of the second treatment (65-min point) and up to four hours afterwards (90-, 120-, 180-, and 240-min points). The results are expressed as relative fluorescence units (RFU) measured at specified time points.

Intracellular ROS production was also evaluated upon 24-h treatment with selected DHPs. Following 24-h treatment, media was replaced with the Hanks’ solution containing 10 μM DCFH-DA and cells were further incubated for 30 min on 37 °C. Next, the medium was replaced with the fresh one and fluorescence was measured prior to the addition of 50 µM tBHP (zero-point) and after 1-h. Measurements were carried with a Cary Eclipse Fluorescence Spectrophotometer (Varian Australia Pty Ltd, Mulgrave, Victoria, Australia) as previously stated. Cells were further trypsinized with 0.25% (*w*/*v*) Trypsin-0.53 mM EDTA solution and counted with Trypan Blue Exclusion Assay in a Bürker–Türk hemocytometer (Brand, Wertheim, Germany). The results are expressed in arbitrary units which are a ratio of the difference in fluorescence (1-h point–zero-point) and cell number.

### 2.8. Determination of Glutathione (GSH) Levels

Cells were first trypsinized (0.25% (*w*/*v*) Trypsin-0.53 mM EDTA solution), washed twice with phosphate-buffer saline (PBS) and stored as dry pellets at −80 °C until analysis. Cell lysates were obtained by addition of 100 µL PBS and repeated thaw-freeze cycles, followed by centrifugation at 16,000× *g*/15 min. and collection of a supernatant containing proteins. Protein concentration in each sample was determined according to Bradford method [33], using bovine serum albumin as a standard.

The total intracellular GSH content (oxidized and reduced) was measured as previously described [34]. Briefly, 150 μL of each sample, containing 0.03 mg/mL of protein, or standard, reduced glutathione in serial dilutions (0–20 mg/mL) was pipetted into each well of 96-microwell plate. Reaction was started by addition of freshly prepared reaction mixture: 1.8 mM 5,5-dithio-bis-2-nitrobenzoic acid, 0.4 U GSH reductase, and 0.6 mM NADPH in phosphate buffer (100 mM NaH_2_PO_4_, 5 mM EDTA pH 7.4). The formation of 2-nitro-5-thiobenzoic acid was monitored spectrophotometrically in a plate reader at 405 nm (Multiscan Ex, Thermo Electron Corporation, Shanghai, China). Total GSH concentration in cell lysates was calculated from the standard curve by linear regression and expressed as µM of total GSH.

### 2.9. Statistical Analysis

Cell-free colorimetric assays (TAC and TOC) were conducted in duplicates while cell-based methods (EZ4U assay, BrdU assay, ROS and GSH measurements) were carried out in at least triplicates. The respective numbers of biological replicates (n) is given in each figure legend. Data are expressed as mean values with standard deviations. Results were analyzed with Student’s *t*-test and values of *p* < 0.05 were considered as statistically significant.

## 3. Results

### 3.1. Total Antioxidative Capacity

The results of the total antioxidative capacity assay for tested DHPs are listed in Table 2. In comparison to uric acid, used as a standard, the following compounds (at 1 mM concentration) were more effective: Diethone **I**, J-9-133-2 **II-2**, AV-153 **IV-1**, AV-154-Na **IV-5**, J-11-71-2 **IV-7**, Carbatone **II-1**, Thiocarbatone **III-1**, E-2-134-1 **III-4**, E-2-136-2 **III-6**, V-6-55-1 **IV-4**, and E-3-46 **IV-3** (about 10-fold or more); J-11-61B **IV-6** (8-fold); J-9-46 II-3 and E-2-135 **III-5** (2-fold). J-9-117 **II-6** and E2-130-3 **III-8** were as effective as uric acid while other tested compounds did not have pronounced antioxidative potential. Accordingly, the DHPs with pronounced antioxidative capacities were selected for the treatment of human osteoblast-like cells (HOS).

### 3.2. Total Oxidative Capacity

The results of the total oxidative capacity assay are listed in Table 3. Oxidative potential of the majority of the tested DHPs was either absent or, if present, not proportional to the concentration used. AV-153 **IV-1**, Carbatone **II-1** and E-170-4 **III-3** (TK1 **III-3**) were the only compounds showing oxidative potential somewhat proportional to the concentration used. While pro-oxidative activity of E-170-4 **III-3** (TK-1 **III-3**) was very low, but proportional to the concentration range, mild oxidative potential of lower concentrations (10 µM and 100 µM) and negligible of the highest (1000 µM) was observed for AV-153 **IV-1** and Carbatone **II-1**. Still, it was about 2-fold (AV-153 **IV-1**) and 5-fold (Carbatone **II-1**) lower than of hydrogen peroxide.

### 3.3. Short-Term (1-h) Treatment of HOS Cells with DHPs and Hydrogen Peroxide

#### 3.3.1. Measurement of Intracellular ROS Production

**Figure 1:**
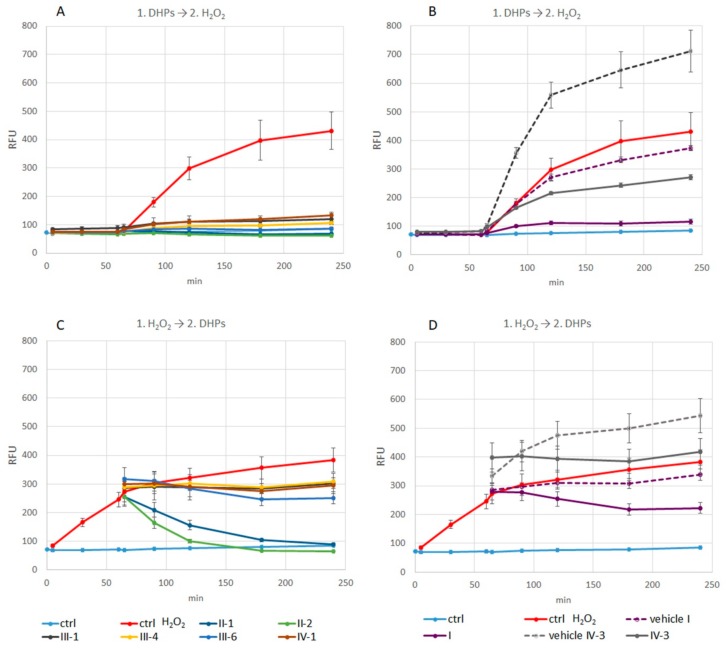
Intracellular ROS of HOS cells exposed to DHPs and H_2_O_2_.Cells were treated with DHPs and hydrogen peroxide, without the exchange of culture medium, as follows: (**A,B**) 1-h DHPs + hydrogen peroxide, or (**C,D**) 1-h hydrogen peroxide + DHPs. The fluorescence reflecting intracellular ROS levels was measured at different time points (0—no treatment, 5—immediately after the first treatment, 30, 60, 65—immediately after the second treatment, 90, 120, 180, 240 min). Results of DHPs (A,C—water-soluble; B,D—soluble in organic solvents) are presented as mean of relative fluorescence units (RFU) ± standard deviation (n = 2). Controls: ctrl (untreated cells), ctrl H_2_O_2_ (cells treated with hydrogen peroxide) and solvent-controls (vehicle Diethone **I** and vehicle E3-46 **IV-3**).

The ability of the tested DHPs to scavenge hydrogen peroxide revealed better reduction of intracellular ROS levels if DHPs were applied before the addition of the stressor. Such antioxidative effects were especially pronounced for water-soluble DHPs and Diethone **I**, observed as reduction of ROS levels almost to level of non-stressed control (Figure 1A,B). The E3-46 **IV-3** was not so effective, its effects were pronounced after 120 min and were not reaching the effects of the other effective DHPs (Figure 1B). On the other hand, if the cells were first exposed to hydrogen peroxide for one hour, the scavenging ability of the majority of tested DHPs was not so pronounced for, still being present (Figure 1C,D). In this type of treatment, E3-46 **IV-3** was the only compound unable to reduce but even increased ROS levels during 4-h measurement (Figure 1D). In both types of treatments, intracellular ROS levels of control cells (without stressor) remained low during the course of 4-h measurement.

Taken together results of both treatments (DHPs before or after stressor) revealed better antioxidative potential of the tested DHPs given before stressor, i.e., when cells were not already damaged by hydrogen peroxide. Moreover, ROS reduction indicated direct scavenging of hydrogen peroxide by the tested DHPs along with possible induction of the cellular protective mechanisms (endogenous antioxidants).

#### 3.3.2. Cellular Viability

**Figure 2:**
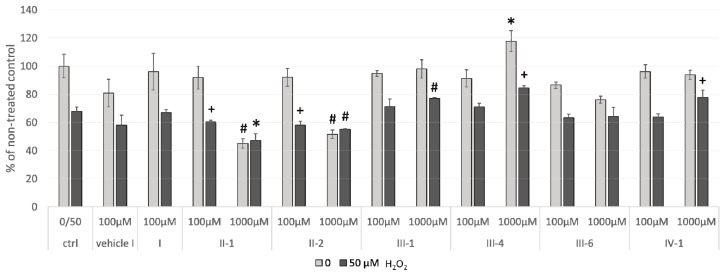
Cellular viability measured by EZ4U assay upon 1 h treatment with DHPs and H_2_O_2_. HOS cells were first treated with DHPs (100 µM and 1000 µM) for one-hour, followed by one-hour treatment with 50 µM hydrogen peroxide or with plain medium. Values are given as mean ± standard deviation; n = 3. Statistically significant differences to their respective controls are shown as follows: * *p* < 0.05; ^+^ *p* < 0.005; ^#^ *p* < 0.0005.

The effects of selected DHPs on cellular viability (Figure 2) were analyzed for cells treated with DHPs alone (non-stress condition) or in combination with 50 µM hydrogen peroxide (IC_30_—corresponding to induction of mild oxidative stress). The E-2-134-1 **III-4** (1000 µM) significantly increased cellular viability both in case of non-stress and in mild-stress culturing conditions (*p* < 0.05 and *p* < 0.005, respectively) while for AV-153 **IV-1** (1000 µM) and Thiocarbatone **III-1** (1000 µM) cellular viability was significantly increased only under mild-stress conditions (*p* < 0.005 and *p* < 0.0005, respectively). Interestingly decreased cellular viability (metabolism) was observed by EZ4U assay for Carbatone **II-1** and J-9-133-2 **II-2**, which were previously (Figure 1) found to reduce ROS levels, even below non-treated control levels.

Therefore, evaluation of ROS production and cellular viability for short-term treatments have accentuated Thiocarbatone **III-1**, AV-153 **IV-1** and in particular E-2-134-1 **III-4** (at 1000 µM concentration) as likely the most potent and bioactive DHP antioxidants in the tested group due to their beneficial growth promoting and ROS scavenging effects under mild oxidative stress conditions.

### 3.4. The Effects of 24-h Pre-Treatment of Cells with DHPs before Exposure to Hydrogen Peroxide or Tert-Butyl Hydroperoxide

#### 3.4.1. Hydrogen Peroxide as a Stressor

**Figure 3:**
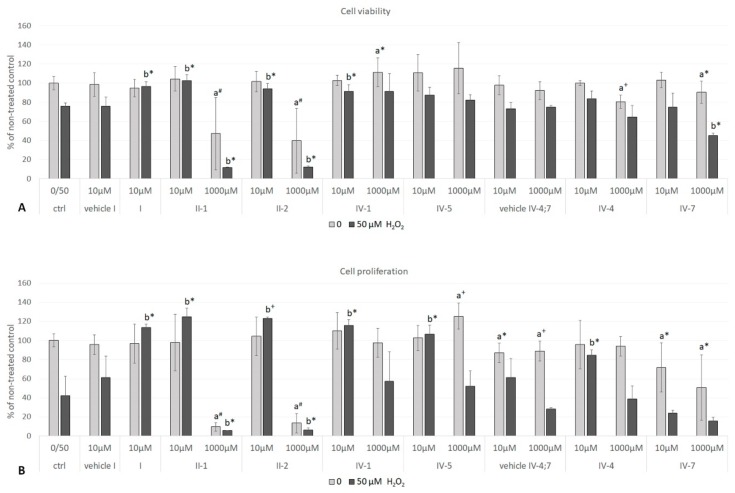
Cell viability (**A**) and proliferation (**B**) upon 24-h treatment with DHPs followed by 1-h exposure to H_2_O_2_. Results are expressed as a percentage of non-treated control. Statistically significant differences between—(a) DHPs alone vs. non-treated control (pale grey bars) or (b) DHPs treated vs. treated control (dark grey bars) are shown as follows: a*/b* *p* < 0.05; a^+^/b^+^ *p* < 0.005; a^#^/b^#^ *p* < 0.0005. Values are given as mean ± standard deviation, n = 4.

The possible growth-promoting potential of DHPs was further evaluated in non-stress (without addition of stressor) as well as in mild oxidative stress conditions (addition of H_2_O_2_ as stressor). Stimulatory effects of 1000 µM AV-153 **IV-1** and AV-154-Na **IV-5** on cell viability (EZ4U assay, *p* < 0.05) or proliferation (BrdU assay, *p* < 0.005), respectively, were observed. Other DHPs did not enhance but have even inhibited cell growth, particularly 1000 µM Carbatone **II-1** and J-9-133-2 **II-2**. Treatment with hydrogen peroxide affected more cell proliferation than viability. Negative effect of hydrogen peroxide was attenuated with low concentration (10 µM) of AV-153 **IV-1**, AV-154-Na **IV-5**, J-9-133-2 **II-2**, Carbatone **II-1** and Diethone **I** (Figure 3A,B).

The results have shown attenuation of oxidative stress-induced suppression of cell growth for AV-153 **IV-1**, AV-154-Na **IV-5**, J-9-133-2 **II-2**, Carbatone **II-1**, and Diethone **I** (when present at low concentrations), thus suggesting potential beneficial effects as of these DHPs as bioactive antioxidants.

#### 3.4.2. Tert-Butyl Hydroperoxide(tBHP) as a Stressor

##### Cell Viability and Proliferation

**Figure 4:**
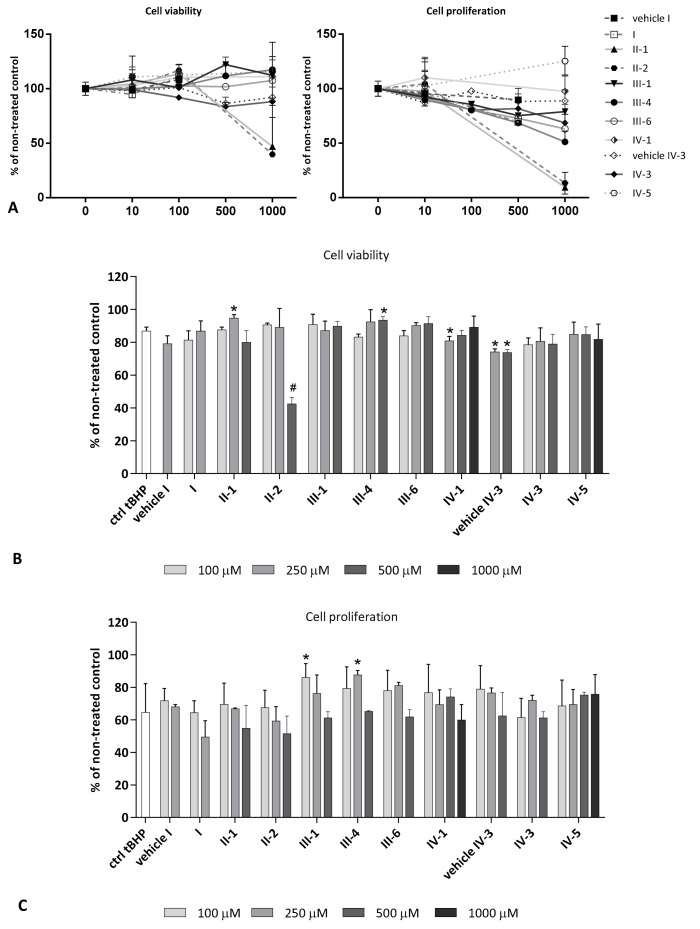
Cell viability (EZ4U assay) and proliferation (BrdU assay). (**A**) just DHPs without tBHP; (**B**) and (**C**) cell cultures treated with 50 µM tBHP. Results are expressed as a percentage of non-treated control. Values are given as mean ± standard deviation; n = 4. In comparison to the treatment control (ctrl tBHP), statistically significant differences are shown as follows: * *p* < 0.05; ^#^ *p* < 0.0005.

Cell viability and proliferation were tested for a range of concentrations (10–1000 µM) of AV-153 **IV-1**, AV-154-Na **IV-5**, J-9-133-2 **II-2**, Carbatone **II-1**, and Diethone **I** (five of previously tested compounds), as well as for Thiocarbatone **III-1**, E2-134-1 **III-4**, E2-136-2 **III-6**, and E3-46 **IV-3** (four newly tested compounds). The majority of selected DHPs, per se, significantly stimulated cellular viability, according to the EZ4U assay, but did not affect cell proliferation according to the BrdU assay (Figure 4A). Hence, different concentrations of DHPs were selected for evaluation of respective DHPs’ effects on HOS cells under mild oxidative stress conditions using 50 µM tBHP as a stressor. As in case of hydrogen peroxide, tBHP suppressed cell proliferation more than cellular viability. The E2-134-1 **III-4** stimulated both, cell viability (500 µM, *p* < 0.05) and proliferation (250 µM, *p* < 0.05) while Carbatone **II-1** (250 µM; *p* < 0.05) and Thiocarbatone **III-1** (100 µM; *p* < 0.05) stimulated either viability or proliferation (Figure 4B,C).

##### Intracellular ROS Production

**Figure 5:**
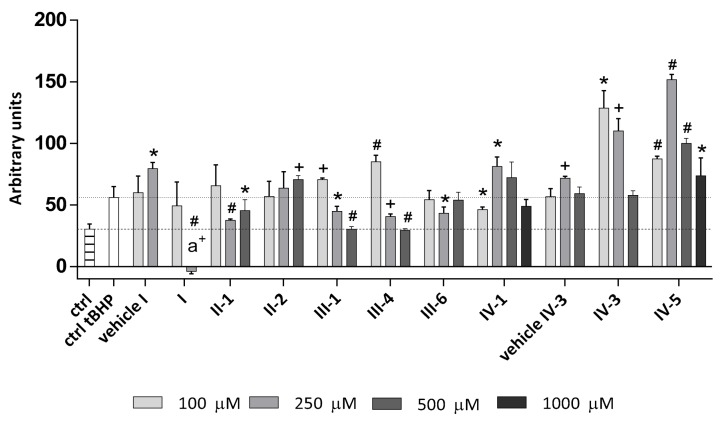
Intracellular ROS levels upon 24-h treatment with DHPs in mild oxidative stress conditions (1-h treatment with 50 µM t-BHP). Results are expressed in arbitrary units, adjusting the difference in fluorescence measurement after 1-h treatment with tBHP and zero-point (before addition of tBHP) with the cell number. Values are given as mean ± standard deviation (*n* = 3). Statistically significant differences are shown as follows: * *p* < 0.05; ^+^ *p* < 0.005; ^#^ *p* < 0.0005 (in comparison to the treatment control (ctrl tBHP; dotted line)); and a^+^ *p* < 0.005 (in comparison to the non-treated control (ctrl; dashed line)).

As expected, pro-oxidant stressor tBHP increased the production of intracellular ROS while DHPs have shown diverse mostly concentration-dependent patterns (Figure 5). The highest reduction of ROS was observed with 250 µM Diethone **I**, which was the only DHP able to decrease ROS levels even below that of non-treated controls (*p* < 0.005). The highest applied concentration (500 µM) of Thiocarbatone **III-1** and of E2-134-1 **III-4**, decreased ROS levels to the level of non-treated controls (*p* < 0.0005). These DHPs were also effective at 250 µM (*p* < 0.05 and *p* < 0.005), while the lowest concentration applied (100 µM) increased intracellular ROS production (*p* < 0.005 and *p* < 0.0005). The AV-153 **IV-1** and AV-154-Na **IV-5** have shown a concentration-dependent bell-shaped curve, whereas in general, AV-154-Na **IV-5** increased ROS levels (suggesting its pro-oxidant role), while similar pro-oxidative effect of AV-153 **IV-1** was just observed in the middle concentration range of the substance used (250 µM; *p* < 0.05), to be followed by antioxidant effect of ROS reduction observed for the highest concentration (100 µM; *p* < 0.05). Carbatone **II-1** was effective in reducing the ROS levels at higher concentrations (250 µM, *p* < 0.0005; 500 µM, *p* < 0.05), while E2-136-2 **III-6** was effective as antioxidant at 250 µM (*p* < 0.05), not affecting ROS levels in other concentrations. Finally, E3-46 **IV-3** and J-9-133-2 **II-2** were mostly effective as pro-oxidants, particularly E3-46 **IV-3** if used at a lower concentration range (100 µM, *p* < 0.05; 250 µM, *p* < 0.005).

##### Determination of Total Glutathione Level in the Cells

**Figure 6:**
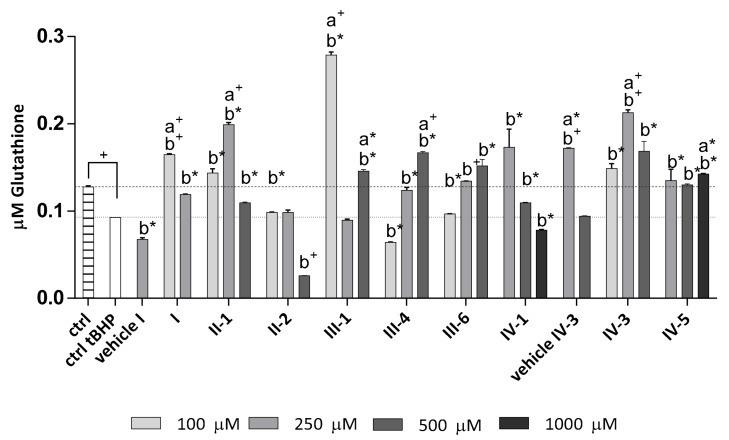
Concentration of total glutathione in cell lysates after 24-h treatment with DHPs followed by 1-h exposure to mild oxidative stress (50 µM tBHP). Statistically significant difference between DHPs and (a) non-treated control (ctrl (dashed line); only for the significant increase) or (b) treated control (ctrl tBHP (dotted line)) is given as follows: a*/b* *p* < 0.05; a^+^/b^+^ *p* < 0.005 with the difference between ctrl vs. ctrl tBHP as ^+^ *p* < 0.005. Values are given as mean ± standard deviation, n = 3.

Glutathione depletion was observed in HOS cells upon tBHP treatment (Figure 6). All tested DHPs were able to amend such tBHP-induced glutathione depletions at least in the case of some used concentrations. The only case of additional decrease of glutathione below the levels reduced by tBHP was observed for AV-153 **IV-1** (1000 µM; *p* < 0.05), E2-134-1 **III-4** (100 µM; *p* < 0.05), and J-9-133-2 **II-2** (500 µM; *p* < 0.005). Opposite to that some of tested DHPs were able to increase glutathione above the level of non-treated control. Such enhancing effect was observed for AV-154-Na **IV-5** (1000 µM; *p* < 0.05), E2-134-1 **III-4** (500 µM; *p* < 0.05), Diethone **I** (100 µM; *p* < 0.05), Carbatone **II-1** (250 µM; *p* < 0.005), E3-46 **IV-3** (250 µM; *p* < 0.05), and Thiocarbatone **III-1** (100 µM; *p* < 0.005 and 500 µM; *p* < 0.05). Surprisingly, Thiocarbatone **III-1**, while being able to increase glutathione more than the other tested DHPs (100 µM; *p* < 0.005), if applied at 250 µM concentration this DHP did not amend the tBHP-caused glutathione-depletion, thus producing the U-shaped concentration-dependence curve. The glutathione levels for other DHPs were either proportional with their increasing concentrations (e.g., E2-134-1 **III-4**, E-2-136-2 **III-6**) or decreasing with their increasing concentrations (e.g., AV-153 **IV-1**, Diethone **I**) or forming a bell-shaped curve (e.g., Carbatone **II-1**, E3-46 **IV-3**).

In summary, the results of 24-h exposure of cells to DHPs prior the induction of mild oxidative stress (50 µM tBHP) revealed that Thiocarbatone **III-1**, Carbatone **II-1** and in particular E2-134-1 **III-4** were the only DHPs able to promote cell growth under oxidative stress conditions, acting in a concentration-dependent manner. While growth promotion was associated, as expected, with reduction of ROS and induction of the intracellular glutathione for E2-134-1 **III-4** (250 µM and 500 µM) and Carbatone **II-1** (250 µM), in case of the most pronounced increase of glutathione observed for Thiocarbatone **III-1** (100 µM), an increase of intracellular ROS was shown, too.

## 4. Discussion

The results of our study are in line with emerging evidence supporting pleiotropic, against oxidative stress-oriented, actions of DHPs. Thus, 4-aryl-2,6-dimethyl-3,5-bis-*N*-(aryl)-carbamoyl-1,4-dihydropyridines is already in use as novel skin-protecting agents, which inhibit elastase enzyme and protect against ROS [35]. In addition, the antioxidative ability of DHPs was used for creating more stable, light-sensitive DHP polythiophene derivatives (PTDHPs) as better alternatives to fluorescent probes used for cell imaging. These PTDHPs, via DHP groups, were found to regulate angiotensin-induced intracellular oxidative stress [36]. Recently, effective ROS radical scavenging was found also for some mitochondria-targeting DHPs [37]. These Mito-DHPs are promising antioxidants since they could protect both against radiation- and ROS-induced DNA strand breaks. These findings further confirm previous data on antioxidant and reductant activities of DHPs (Diethone **I** and its analogues) [38,39], pointing to mitochondria as targets for cell protective, bioactive DHP antioxidants [40,41]. This assumption is further supported by the current study since we observed differences in biological activities of DHPs in respect to biological parameter analyzed (cell proliferation vs. viability), which might be due to the fact that cellular viability was analyzed by EZ4U assay that reflects mitochondrial dehydrogenase activities. The same stands also for the observed differential effects of DHPs in respect to their direct pro- and anti-oxidative capacities (TOC/TAC assays based on hydrogen peroxide/peroxidase activity principle) vs. influence of DHPs on intracellular production of ROS, which is dependent on cellular oxidative homeostasis affecting viability and mitochondrial stability of the cells.

This study of pro- and anti-oxidant capacities of 27 structurally different DHPs (as well as one pyridine type compound) support previous findings, further indicating growth-regulating bioactivities of several DHPs acting on human osteoblast-like cells. The used DHPs, roughly divided into four subtypes comprising Diethone (Diludine) **I** and its analogues, Carbatone **II-1** and its analogues, Thiocartbatone (**III-1**) and its analogues, as well 1,4-dihydroisonicotinic acid derivatives (such as AV-153 **IV-1**) and its analogues, were primarily evaluated for their antioxidative ability (TAC assay) as well as for their potential pro-oxidative ability (TOC assay). Antioxidative potential of 1 mM concentration of Diethone **I**, AV-154-Na **IV-5**, Thiocarbatone **III-1**, E2-134-1 **III-4**, E2-136-2 **III-6**, J-9-133-2 **II-2**, E3-46 **IV-3**, J-11-71-2 **IV-7**, V-6-55-1 **IV-4**, Carbatone **II-1**, and AV-153 **IV-1** was about 10-fold higher than was antioxidant capacity of uric acid, the well-known natural antioxidant used as standard. There are main, node structures of DHPs possessing quite high antioxidative capacity known for Diethone **I**, Carbatone **II-1**, AV-153 **IV-1** and AV-154-Na **IV-5**, according to the structure: function analysis. Extension of substituents in positions 3 and 5 of Diethone **I** (ethoxycarbonyl to alkoxycarbonyl-methoxycarbonyl) and its thioderivative (E170-4, TK-2 **III-2**) leads to substantial diminution of antioxidative capacity. On the contrary, compounds possessing carboxylate anions in positions 3 (or 3 and 5) of Diethone **I** possess high antioxidative activity (Carbatone **II-1**, Thiocarbatone **III-1**, also 4-carboxycarbatone V-6-55-1 **IV-4**, hybrids of Carbatone **II-1** or Thiocarbatone **III-1** and Diethone **I** such as E2-134-1 **III-4**, J-9-133-2 **II-2**). Insertion of substituents in position 4 of Carbatone **II-1** (methyl, ethyl, styryl groups) diminishes antioxidative activity to the levels of uric acid for Styrylcarbatone **II-6** (J-9-117 **II-6**) and Metcarbatone **II-4** or almost completely suppresses in case of Etcarbatone **II-5**. However, a carboxylate anion in position 4 leads to the already mentioned antioxidative activity observed for AV-153 **IV-1** and AV-154-Na **IV-5**. Moreover, hybrids of Carbatone **II-1** ester and Diethone **I** possess high antioxidative capacity if compounds have 4-carboxylate anion (V-6-55-1 **IV-4** and E3-46 **IV-3**). If carboxylate groups in position 4 are distant from the DHP cycle (Glutapyrone **IV-8**), antioxidative activity is absent. The oxidized form (pyridine type compound J-12-25 **V**) was found to be inactive. Antioxidant activity mechanisms of DHPs studied may include direct scavenging of ROS (namely, hydrogen peroxide and free radicals derived from it) and decomposition of hydrogen peroxide in the manner, in which ROS will be produced in lesser degree. Namely, it was observed before that Carbatone **II-1** and its derivatives scavenge hydroxyl radicals (spectrophotometric and EPR detection) produced in Fenton reaction [7,14]. Furthermore, EPR spectroscopy showed that Metcarbatone **II-4** acts as effective scavenger of hydroxyl radicals produced in the Fenton reaction, while Etcarbatone **II-5**, and Propcarbatone (close analogue of Etcarbatone **II-5** having propyl group instead of ethyl group) are less effective, and Styrylcarbatone **II-6** is not effective at all. In addition, DHPs affecting TAC could be converted in their heteroaromatic oxidized form [42].

Pro-oxidative potential, measured by the TOC assay (Table 3), of the majority of the tested DHPs was either absent or minor. AV-153 **IV-1**, Carbatone **II-1** and E-170-4 **III-3** (TK1, **III-3**) were the only compounds showing oxidative potential somewhat proportional to the concentration used. While pro-oxidative activity of E-170-4 **III-3** (TK-1 **III-3**; diethyl ester of Thiocarbatone **III-1**) was very low, mild oxidative potential of lower concentrations (10 µM and 100 µM) and negligible of the highest (1000 µM) were observed for AV-153 **IV-1** and Carbatone **II-1**. Still, it was about 2-fold (AV-153 **IV-1**) and 5-fold (Carbatone **II-1**) lower than of hydrogen peroxide. Therefore, the oxidative ability tested by this assay was very moderate or absent suggesting that DHPs are less prone to oxidative activity. Concerning TOC effects, it should be mentioned that Tirzit et al. [43] showed possibility of free-radical reaction propagation by DHPs in vitro. If water-soluble DHPs (where R^4^ = H, or CH_3_, or COONa) have methyl group in the 4-th position, the possibility of that compounds reactivity against hydroxyl radical generation is lowered. However, introduction of strong electron donor group carboxylate-ion (COONa) causes sharp increase of reaction power. Even the introduction of carboxylate-ion in the positions 2 and 6 of the DHP ring increased this reactivity. The generation potency was observed at 0.5 mM and 1 mM, respectively 500 μM and 1000 μM concentrations, while at higher concentrations, the hydroxyl radical (HO•) generation is more pronounced. In addition, both assays, TAC and TOC, use HRP (horse radish peroxidase)-catalyzed oxidation of 3,3′,5,5′-tetramethylbenzidine (TMB) (reduced form of this is colorless) which includes either free radical (one-electron oxidation product) and/or charge transfer complex intermediates, both resulting in the formation of blue-colored reaction product [44]. However, it is difficult to discriminate between a one-electron and a two-electron mechanism for the initial enzymatic step in the reaction, thus the DHPs could react even as free radical quenchers or propagators (by one-electron or two-electron mechanisms) of free radical chain reactions (depending on assays used, DHPs chemical structure and concentration). Indeed, both, strong antioxidative activity and mild oxidative potential was observed for Carbatone **II-1** and AV-153 **IV-1**. Moreover, as being effectors of TAC/TOC systems in the presence of HRP, 1,4-DHPs themselves could also be enzymatically oxidized [45,46]. Thus, the elementary steps of oxidation of water-insoluble lipophilic calcium antagonist Nifedipine (NF) catalyzed by enzyme HRP have been described by analysis of kinetic magnetic field effects (MFEs). It has been shown that the first step of the catalytic cycle is single electron transfer resulting in formation of NF*(+) radical cation and ferroperoxidase (Per^(2+)^) [46]. As a result, comparison with an earlier studied oxidation reaction of NADH catalyzed by HRP evidenced that the enzymatic oxidations of two substrates, native, NADH, and its synthetic DHP analogue, NF-catalyzed by HRP in the absence of hydrogen peroxide follow identical mechanisms. It is likely, that analogous reaction route could proceed in the case of the set of lipophilic DHPs (see Table 1) studied here. Besides, peroxide oxidation (without peroxidase, in Fenton system a.o.) of DHPs could occur [47].

Since previous studies have shown that various DHPs can achieve modulation of cellular responses to oxidative stress and thus influence cell growth and differentiation [30,48], the major aim of the current study was to find a DHP compound or a group of them that will act as potential antioxidants and could modify the growth of human–osteoblast cells indicating their possibly beneficial effects for treatment of osteoporosis and related bone disorders. Since oxidative stress was implicated in pathogenesis of osteoporosis (manifested as deterioration of bone) and antioxidants, such as lycopene and polyphenols have been suggested to benefit the therapy of osteoporosis [17,19,49], we studied the antioxidative potential of the selected DHPs which were 10-fold more effective than uric acid on HOS cells in mild oxidative stress conditions. Although normal human osteoblasts would be preferred model, due to their limited availability and donor-related influences, human osteoblast-like cancer cell lines have been widely used model systems for human osteoblasts, in particular HOS cells are known as such in vitro model [20,21,22].

To evaluate short-term, cell-protective (ROS decreasing) effects of DHPs, cells were treated with the selected DHPs and hydrogen peroxide, in general, for 1-h each. Thiocarbatone **III-1**, AV-153 **IV-1**, E2-134-1 **III-4**, E2-136-2 **III-6**, and Diethone **I** decreased ROS levels showing antioxidative potential, which was pronounced if the cells were not already damaged by hydrogen peroxide. Moreover, observed ROS reduction indicates direct scavenging of hydrogen peroxide by the mentioned DHPs along with possible induction of cellular-protective, antioxidant mechanisms. In addition, while Diethone **I** and E2-136-2 **III-6** did not affect cell growth, Thiocarbatone **III-1**, AV-153 **IV-1** and in particular E2-134-1 **III-4** did, thus emerging as the most potent antioxidants in the tested group, able to reduce oxidative stress-induced damage. Noteworthy, we cannot state with certainty whether the observed beneficial effects upon short-term treatment (DHPs and hydrogen peroxide being present in the medium at the same time for specified time-points) are just due to scavenging of hydrogen peroxide by DHPs (which is negligible without added catalysts) or their ability to induce cellular protective mechanism(s), or even both.

We further explored whether DHPs can induce cellular protective mechanism(s) under mild oxidative stress conditions and if longer exposure of cells to DHPs can potentiate possible effects. To do so, cells were treated with the selected DHPs for 24-h which were removed from the medium prior to exposure to stressors (1-h treatment). Hydrogen peroxide and tert-butyl hydroperoxide (tBHP) were used for induction of mild oxidative stress. In addition to the evaluation of cell growth, measurements of ROS generation along with intracellular glutathione content were carried out with the latter (tBHP) since it is known as more stable oxidative stress-inducing agent than hydrogen peroxide that provides more consistent effects [50]. Notably, for HepG2 cells tBHP, but not hydrogen peroxide, was found to be able to decrease reduced glutathione as wells as to increase malondialdehyde (MDA) levels and activity of antioxidative enzymes [50]. Moreover, tBHP was shown to induce caspase-dependent apoptosis in endothelial cells, which was mediated by ROS generation deriving from NADPH oxidase and mitochondria [51]. However, we should also stress the fact that hydrogen peroxide in very important natural ROS with multiple positive and negative bioactivities.

Both used stressors decreased cell growth, thus creating mild oxidative stress, while AV-153 **IV-1**, AV-154-Na **IV-5**, J-9-133-2 **II-2**, Carbatone **II-1**, and Diethone **I** (at low concentration) ameliorated negative effects of hydrogen peroxide, by promoting cell growth and acting as antioxidants.

The more in-depth analysis of the selected DHPs, considering the influence on cell growth, ROS production and glutathione content, was carried out with tBHP, known to be more stable and consistent in induction of oxidative stress, unlike hydrogen peroxide, which has multiple biological activities and is rapidly metabolized as natural ROS. Glutathione is one of the most important intracellular antioxidants able to scavenge ROS thus becoming oxidized. The increase in ratio oxidized/reduced glutathione indicates severity of oxidative stress. tBHP is known to increase this ratio thus causing depletion of reduced glutathione [50]. We measured total glutathione content (oxidized + reduced) which should be influenced only by de novo synthesis. tBHP decreased glutathione content, also observed in Rat clone-9 hepatocytes [52] which could be due to attenuated activation of the GCLc (glutamate cysteine ligase, catalytic subunit) promoter [53], resulting in reduced biosynthesis.

The selected DHPs were able to amend tBHP-induced glutathione depletions at least in some of the tested concentrations. Such effect was observed in rats exposed to benzo(a)pyrene-induced oxidative stress, where pre-treatment with Nitrendipine increased the glutathione but also superoxide dismutase (SOD) levels while decreasing TBARS levels [54].

Moreover, AV-154-Na **IV-5** (1000 µM), E2-134-1 **III-4** (500 µM), Diethone **I** (100 µM), Carbatone **II-1** (250 µM), E3-46 **IV-3** (250 µM), and Thiocarbatone **III-1** (100 µM and 500 µM) were able to induce glutathione biosynthesis. Indeed, the concentration-dependent diversity in patterns of intracellular glutathione and of ROS levels was observed in our study. Yet, Thiocarbatone **III-1**, Carbatone **II-1** and in particular E2-134-1 **III-4** were the only DHPs able to promote cell growth under such mild oxidative stress conditions acting in a concentration dependent manner. While growth promotion was related to the reduction of ROS and the induction of intracellular glutathione for E2-134-1 **III-4** (250 µM and 500 µM) and Carbatone **II-1** (250 µM), with the strongest observed increase of glutathione, the increase of intracellular ROS was also noticed for Thiocarbatone **III-1** (100 µM).The major pathway activated upon stress conditions is NRF2/KEAP1 [55]. NRF2 activates genes that encode phase II detoxifying enzymes and antioxidant enzymes thus influencing glutathione content. Recently, DHPs (Nifedipine and Amlodipine) were shown to activate NRF2 via a phosphoinositide 3-kinase (PI3K)-dependent mechanism [56]. At this point, we cannot fully explain observed concentration-dependent diversities in ROS and glutathione content, being sometimes reversal as expected, and the others not, or can we explain lack of linearity. However, we can assume that the onset of lipid peroxidation, generating reactive aldehydes, especially 4-hydronynoenal known to act as a second messenger of free radicals and signaling molecules regulating cell growth and inducing endogenous production of ROS, eventually affecting the activity and synthesis of glutathione might be, at least in part, responsible for that [57,58,59]. Interestingly, these DHPs did not affect cell growth, unlike growth-supporting DHPs E2-134-1 **III-4** and Carbatone **II-1**, which were found to increase glutathione and reduce ROS production, thus acting as potent antioxidants. On the other hand, Thiocarbatone **III-1** could not decrease ROS although it enhanced the growth of the HOS cells. These findings again suggest involvement of other survival pathway(s) that might include second messengers of free radicals, which are since recently also in focus of research as growth regulators [60,61,62]. In favor of this possibility are also pro-oxidative effects of Thiocarbatone **III-1**, resembling similar effects of HNE, while the other DHPs were also acting as pro-oxidants at certain concentration.

This dual phenomenon is characteristic also for some other compounds (e.g., ascorbic acid), acting either as antioxidants or as pro-oxidants, depending on given conditions [63]. For alpha-tocopherol (vitamin E) and even for polyphenols and thiols, the persistent rhetoric question is if vitamin E should be considered as a pro- or an anti-oxidant [64,65]. This question is actual similar for DHPs, in the case of which the likely answer could be both antioxidants and/or pro-oxidants, depending on the compound’s individual structure determinants, like C. Winterburn stated: “Antioxidants are individuals” [65]. The same appears to be valid for bioactive DHP antioxidants, too.

DHPs, such as Thiocarbatone **III-1**, E2-134-1 **III-4**, Carbatone **II-1**, AV-153 **IV-1**, AV-154-Na **IV-5**, J-9-133-2 **II-2**, and Diethone **I** were able to attenuate negative effects of oxidative stress in human osteoblast-like cells. Their action was highly dependent on the concentration used, stressor applied and treatment set-up. Thus, the observed growth-supporting effects suggest that they might be eventually considered as promising therapeutic agents in the treatment of osteoporosis as was observed for Cilnidipine [66] and Amlodipine [67] in murine translation models.

## 5. Conclusions

Eleven out of the 28 structurally different compounds tested (27 DHP derivatives and one oxidized form pyridine-type heteroaromatic compound), in particular, Diethone **I,** AV-154-Na **IV-5**, Thiocarbatone **III-1**, E2-134-1 **III-4**, E2-136-2 **III-6**, J-9-133-2 **II-2**, E3-46 **IV-3**, J-11-71-2 **IV-7**, V-6-55-1 **IV-4**, Carbatone **II-1** and AV-153 **IV-1** were about 10-fold more effective antioxidants than uric acid and were further tested in the HOS model of human osteoblast-like cells. From the selected DHPs, Thiocarbatone **III-1**, E2-134-1 **III-4**, Carbatone **II-1**, AV-153 **IV-1**, AV-154-Na **IV-5**, J-9-133-2 **II-2**, and Diethone **I** were able to attenuate negative effects of oxidative stress in human osteoblast-like cells induced by pro-oxidants. These bioactive DHP antioxidants acted in a concentration-dependent manner, while their efficiency was also related to the pro-oxidant stressor applied and experimental treatment set-up. Thiocarbatone **III-1**, Carbatone **II-1** and E2-134-1 **III-4** were found to be undoubtedly the most potent compounds with growth-promoting effects and the only DHPs being able to act so in tBHP-induced oxidative stress conditions. The observed induction of de novo glutathione synthesis by HOS cells, observed upon treatment with these DHPs, implicates involvement of the NRF2 signaling pathway. While Carbatone **II-1** and E2-134-1 **III-4** have shown concordant reduction in ROS, for Thiocarbatone **III-1**, increased ROS contributing to cell growth suggested other survival mechanism(s) involved. Surprisingly, Diethone **I** (known antioxidant) did not affect cell growth even though it decreased ROS and mostly resulted in increasing glutathione in such conditions. Likewise, Diethone **I** and AV-153 **IV-1** were more protective if hydrogen peroxide was used as a stressor. In addition, AV-153 **IV-1** appeared to be more effective if directly acting as a scavenger of hydrogen-peroxide for shorter time, like Diethone **I**, while Thiocarbatone **III-1** and E2-134-1 **III-4** acted seemingly both as scavengers of ROS and as inducers of growth promoting signaling pathways. Therefore, we assume that HNE and related second messengers of free radicals generated by lipid peroxidation might be relevant factors for the bioactivity principles of bioactive DHPs, which should be further studied.

Finally, the results of our study together with supporting evidence for other DHPs [66,67] as well as with other antioxidants [17], suggest that DHPs could be considered as therapeutic agents for osteoporosis although further research is needed to elucidate mechanisms involved.

## Figures and Tables

**Table 1:**
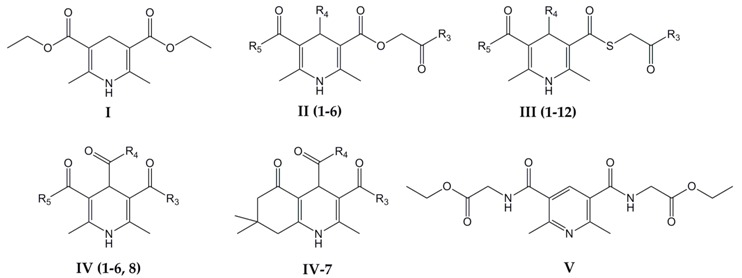
Chemical structures and solubility of 27 1,4-dihydropyridine derivatives and one pyridine analogue investigated in this study.

Compounds	Trivial Name	R3	R4	R5	Solubility	Reference
**I**	Diethone				Ethanol with 10% DMSO	[23]
**II-1**	Carbatone	ONa	H	OCH_2_COONa	Water	[24]
**II-2**	J-9-133-2	ONa	H	OCH_3_	Water	[25]
**II-3**	J-9-46	OCH_2_CH_3_	H	OCH_2_CH_3_	Ethanol	[25]
**II-4**	Metcarbatone	ONa	CH_3_	OCH_2_COONa	Water	[26]
**II-5**	Etcarbatone	ONa	CH_2_CH_3_	OCH_2_COONa	Water	[26]
**II-6**	J-9-117 (Styrylcarbatone)	ONa	CH=CHPh	OCH_2_COONa	Water	*MM
**III-1**	Thiocarbatone	ONa	H	SCH_2_COONa	Water	MM
**III-2**	TK-2	OCH_3_	H	SCH_2_COOCH_3_	Ethanol with 10% DMSO	MM
**III-3**	E-170-4 (TK-1)	OCH_2_CH_3_	H	SCH_2_COOCH_2_CH_3_	Ethanol with 10% DMSO	MM
**III-4**	E2-134-1	ONa	H	OCH_2_CH_3_	Water	MM
**III-5**	E2-135	OCH_2_CH_3_	H	OCH_2_CH_3_	Ethanol	MM
**III-6**	E2-136-2	ONa	H	OCH_2_COONa	Water	MM
**III-7**	E2-131	ONa	CH_2_CH_3_	OCH_2_CH_3_	Water	MM
**III-8**	E2-130-3	OCH_2_CH_3_	CH_2_CH_3_	OCH_2_CH_3_	Ethanol	MM
**III-9**	E2-120 (ETK-2)	OCH_3_	CH_2_CH_3_	SCH_2_COOCH_3_	Ethanol	MM
**III-10**	E2-113 (ETK-1)	OCH_2_CH_3_	CH_2_CH_3_	SCH_2_COOCH_2_CH_3_	Ethanol	MM
**III-11**	E-163-1	ONa	3-Py	OCH_2_CH_3_	Water	MM
**III-12**	E-163-K	OCH_2_CH_3_	3-Py	OCH_2_CH_3_	Ethanol	MM
**IV-1**	AV-153	OCH_2_CH_3_	ONa	OCH_2_CH_3_	Water	[27]
**IV-2**	EE-126	OCH_3_	ONa	OCH_3_	Water	MM
**IV-3**	E3-46	OCH_2_CH_3_	OH	OCH_2_COOCH_2_CH_3_	Ethanol	MM
**IV-4**	V-6-55-1	OCH_2_COOCH_2_CH_3_	OH	OCH_2_COOCH_2_CH_3_	Ethanol	MM
**IV-5**	AV-154-Na	CH_3_	ONa	CH_3_	Water	[27]
**IV-6**	J-11-61B	CH_3_	OH	OCH_2_CH_3_	Ethanol	MM
**IV-7**	J-11-71-2	OCH_2_CH_3_	OH		Ethanol	MM
**IV-8**	Glutapyrone	OCH_2_CH_3_	NHCH(CH_2_)_2_COONa|COONa	OCH_2_CH_3_	Water	[28]
**V**	J-12-25				Ethanol	MM

*MM—see in Materials and Methods. DMSO (Dimethyl sulfoxide).

**Table 2:** Total antioxidant capacity of selected compounds expressed as equivalent to mM uric acid.

Compounds	Trivial Name	Concentrations Tested		
		10 µM	100 µM	1000 µM
		Equivalent to mM Uric Acid		
**I**	Diethone	1.463 ± 1.016	1.465 ± 0.085	>10
**II-1**	Carbatone	0.438 ± 0.177	1.534 ± 0.064	9.692 ± 0.036
**II-2**	J-9-133-2	0.585 ± 0.074	3.756 ± 0.312	>10
**II-3**	J-9-46	0	0.148 ± 0.088	2.284 ± 0.504
**II-4**	Metcarbatone	0	0.526 ± 0.033	0.852 ± 0.013
**II-5**	Etcarbatone	0.027 ± 0.038	0.057 ± 0.019	0.206 ± 0.085
**II-6**	J-9-117 (Styrylcarbatone)	0.680 ± 0.051	0.421 ± 0.596	1.073 ± 0.04
**III-1**	Thiocarbatone	1.279 ± 0.046	2.735 ± 0.363	>10
**III-2**	TK-2	0	0	0
**III-3**	E-170-4 (TK-1)	0	0	0
**III-4**	E2-134-1	0.770 ± 0.075	0.541 ± 0.304	>10
**III-5**	E2-135	0.027 ± 0.302	0.564 ± 0.422	1.968 ± 0.294
**III-6**	E2-136-2	0.498 ± 0.115	1.082 ± 0.175	>10
**III-7**	E2-131	0.778 ± 0.085	0	0
**III-8**	E2-130-3	0.355 ± 0.234	0.285 ± 0.398	0.980 ± 0.129
**III-9**	E2-120 (ETK-2)	0	0.042 ± 0.007	0
**III-10**	E2-113 (ETK-1)	0.032 ± 0.195	0.077 ± 0.219	0
**III-11**	E-163-1	0	0	0
**III-12**	E-163-K	0	0	0
**IV-1**	AV-153	0.153 ± 0.216	2.005 ± 0.053	9.828 ± 0.081
**IV-2**	EE-126	0.856 ± 0.614	0.304 ± 0.078	0.292 ± 0.083
**IV-3**	E3-46	0.075 ± 0.163	1.542 ± 0.120	>10
**IV-4**	V-6-55-1	0.636 ± 0.220	4.013 ± 0.228	>10
**IV-5**	AV-154-Na	0.481 ± 0.028	2.774 ± 0.199	>10
**IV-6**	J-11-61B	0	0.948 ± 0.056	8.222 ± 0
**IV-7**	J-11-71-2	0	3.160 ± 0.094	>10
**IV-8**	Glutapyrone	0.190 ± 0.015	0.127 ± 0.096	0
**V**	J-12-25	0.016 ± 0.193	0.050 ± 0.040	0.012 ± 0.044

**Table 3:** The oxidative capacity of selected compounds expressed as equivalent to µM H_2_O_2_.

Compounds	Trivial Name	Concentrations Tested		
		10 µM	100 µM	1000 µM
		Equivalent to µM H_2_O_2_		
**I**	Diethone	1.346 ± 1.904	0	0
**II-1**	Carbatone	2.692 ± 0.000	18.846 ± 1.088	1.538 ± 0.544
**II-2**	J-9-133-2	0.250 ± 0.374	0	0
**II-3**	J-9-46	0	0	0
**II-4**	Metcarbatone	0	0	0
**II-5**	Etcarbatone	0	0	0
**II-6**	J-9-117 (Styrylcarbatone)	0.577 ± 0.816	0	0.192 ± 0.272
**III-1**	Thiocarbatone	0	0	0
**III-2**	TK-2	0	0	7.558 ± 1.791
**III-3**	E-170-4 (TK-1)	0	3.375±1.768	38.529 ± 1.663
**III-4**	E2-134-1	0	0	0
**III-5**	E2-135	0	0	0
**III-6**	E2-136-2	0	0	0
**III-7**	E2-131	0	5.411 ± 1.663	6.588 ± 3.328
**III-8**	E2-130-3	0	0	0
**III-9**	E2-120 (ETK-2)	0	0	13.235 ± 5.407
**III-10**	E2-113 (ETK-1)	0	0	0.147±0.208
**III-11**	E-163-1	0	4.5 ± 1.179	13.059 ± 1.664
**III-12**	E-163-K	0	0	0
**IV-1**	AV-153	6.923 ± 0.544	41.154 ± 1.088	5.00 ± 4.351
**IV-2**	EE-126	3.625 ± 0.884	3.938 ± 0.442	3.938 ± 0.442
**IV-3**	E3-46	0	7.243 ± 0.114	0
**IV-4**	V-6-55-1	0.481 ± 0.680	0	0
**IV-5**	AV-154-Na	3.938 ± 0.442	3.625 ± 0.884	0
**IV-6**	J-11-61B	0	0	0
**IV-7**	J-11-71-2	0.938 ± 0.000	1.875 ± 0.000	0
**IV-8**	Glutapyrone	0	0	0
**V**	J-12-25	0	0	0

## Abbreviations

DHP(s)	1,4-dihydropyridine(s)
OS	oxidative stress
AO	Antioxidant
AOA	antioxidative activity
TAC	total antioxidative capacity
TOC	total oxidative capacity
ROS	reactive oxygen species
HRP	horse radish peroxidase
TMB	3,3′,5,5′-tetramethylbenzydine
DCFH-DA	2′,7′-dichlorodihydrofluorescein diacetate
tBHP	tert-butyl hydroperoxide
BrdU	5-bromo-2′-deoxyuridine
HOS	human osteosarcoma.
